# Facemask Wearing Among Chinese International Students From Hong Kong Studying in United Kingdom Universities During COVID-19: A Mixed Method Study

**DOI:** 10.3389/fpsyt.2021.673531

**Published:** 2021-06-16

**Authors:** Agnes Yuen-kwan Lai, Shirley Man-man Sit, Theresa Tze-kwan Lai, Man-ping Wang, Cecilia Hoi-mei Kong, Jessica Yuet-ying Cheuk, Yibin Feng, Mary Sau-man Ip, Tai-hing Lam

**Affiliations:** ^1^School of Nursing, The University of Hong Kong, Hong Kong, China; ^2^School of Public Health, The University of Hong Kong, Hong Kong, China; ^3^School of Health Sciences, Caritas Institute of Higher Education, Hong Kong, China; ^4^School of Chinese Medicine, The University of Hong Kong, Hong Kong, China; ^5^Department of Medicine, The University of Hong Kong, Hong Kong, China

**Keywords:** facemask, international students, University students, stress, mental health, Hong Kong, United Kingdom, prejudiced attitudes

## Abstract

**Background:** The mental health of international students studying abroad has been neglected during the COVID-19 pandemic.

**Objective:** This mixed-method study examined perceived public attitudes, personal beliefs, practice and stress toward facemask wearing as a preventive measure against COVID-19 among international University students from Hong Kong studying in the United Kingdom (UK) in the early stage (January–March 2020) of the pandemic.

**Methods:** Our study included 2 parts: (i) an exponential, non-discriminative snowball sampling strategy was used to recruit 91 Chinese students studying in the UK to complete an online questionnaire survey, and (ii) online Zoom focus group interviews were conducted with 16 students who completed the online survey to gain an in-depth understanding of their experiences and coping methods during the pandemic.

**Results:** Of the 91 students, 92.3% reported the UK public did not view facemask wearing as a preventive measure. 98.9% believed facemask wearing was an effective preventive measure, but 56% wore facemasks more than half of the time when out in public. 50.5% had internal conflicts of stress both when wearing and not wearing facemasks, which was more common in females than males [(62.5 vs. 31.5%), *P* = 0.004, Relative Risk (RR): 1.99 (1.17, 3.38)]. 61.5% reported public prejudiced attitudes against facemask wearing, also more common in females than males (71.4 vs. 45.7%), *P* = 0.02, RR: 1.56 (1.05, 2.32). The qualitative findings corroborated with the quantitative findings and reported that peer and family support were important for them to face such difficulties, and positive thinking and adaptability were effective methods on stress management.

**Conclusions:** Since the outbreak of COVID-19, Chinese international students have been faced with a difficult, confusing, and sensitive situation. Owing to the ongoing pandemic, rising xenophobia and racist behaviors and the resumption of students' studies studies in the U.K., support from global communities are needed in their pursuit of quality education overseas. Our findings have significant implications on the proactive roles that governments should have, and the need for clear and accurate public health messaging to change public attitudes and mitigate prejudice. Academic institutions and mental health professionals need to proactively provide additional support to Chinese international students.

## Introduction

The novel coronavirus (COVID-19) pandemic has adversely impacted mental health. Asian communities in Western countries, such as the United Kingdom (U.K.), have been targets of incidents and attacks involving xenophobia, racism and discrimination (1). Wearing facemasks (either cloth or surgical masks) has often been a catalyst for such incidents (2, 3), a phenomenon dubbed as “maskaphobia.” For Chinese international students studying abroad, this problem is especially concerning, but the impacts on their mental health have been largely overlooked (4).

The pandemic has presented unprecedented challenges for global higher education. According to the United Nations Educational, Scientific and Cultural Organization (UNESCO), more than 166 countries have implemented nationwide closures since March 2020, impacting close to 90% of the world's student population (5). From January to March 2020, confirmed COVID-19 cases in the UK increased dramatically to a higher level (*n* = 29,681) than that in Hong Kong (HK) (*n* = 680) (6, 7).

Hong Kong had almost 100% voluntary masking since the end of January 2020 (8). Local public health professionals had been advocating for universal mass masking that has become ubiquitous and is perceived as a civic responsibility and symbol of social solidarity in response to the pandemic (9, 10).

In the U.K., there was no culture and history of wearing facemask. Facemasks were not recommended in Western countries until several months later when outbreaks became uncontrollable. Moreover, the U.K. Prime Minister announced on 12 March 2020 that individuals with a continuous cough or high temperature should self-isolate at home for at least 7 days before seeking medical advice (11). A study conducted in April 2020 also found that only 19% of the U.K. public were wearing facemasks (12). Such policies, low facemask wearing rate and escalating outbreaks in the UK resulted in many international students from Hong Kong returning home where the outbreaks were under better control. Compulsory quarantine orders on all persons arriving from foreign places were imposed in Hong Kong and commenced on 19 March 2020 (13).

Rising xenophobia, specifically Sinophobia, during the pandemic has also been a factor in the decline of Chinese students studying in the U.K. (14) where Chinese students make up the majority of overseas students (15, 16). Discriminatory behaviors can contribute to students' ongoing stress which can negatively affect academic performance and mental well-being (17, 18).

Increasing severity of the outbreak affecting daily life and learning, compounded with growing fears over prejudice and discrimination, including from those against facemask wearing and the emergence of sinophobic behavior online during COVID-19 (19), has likely affected the mental health of international students studying abroad.

Besides, findings from a report of American Psychological Association on 1,134 participants showed that females were more likely than males to report a great deal of stress (8, 9, or 10 on a 10-point scale) and more physical and emotional symptoms of stress (20).

We used a mix-method approach to examine students perceived public attitudes, personal beliefs, practices, stress and stressors in relation to facemask wearing, and explored any differences in stress in relation to facemask wearing and facemask-related stressors between males and females. We hypothesized that females would experience greater stress from facemask wearing than males.

## Methods

### Study Design

This study used different types of information and communication technology (ICT) strategies throughout the process from promotion and recruitment (by instant messaging platform “WhatsApp”) to collection of quantitative (by online survey platform “Qualtrics”) and qualitative data (by video conferencing “Zoom” focus group interviews).

We first conducted a cross-sectional online questionnaire survey on the psychological impact of COVID-19 on University students. Firstly, a link was disseminated through WhatsApp to University students studying in Hong Kong or overseas. These students were encouraged to forward the survey link to their friends. The inclusion criteria were current students aged 18 years or older. In this present paper, additional inclusion criteria were Chinese international students from Hong Kong and studying in U.K. universities. Secondly, for the recruitment of focus group interviews, we encouraged students who completed the survey to provide their phone numbers for further contact. We then invited those students to join the online focus group interviews *via* WhatsApp messages and calls. We used Zoom, a cloud-based video conferencing service for the interviews. An interview link was sent to each participant *via* WhatsApp.

The survey was conducted from 28 April through 12 May 2020. Informed consent was obtained prior to starting the survey. Participation in the survey and interviews was voluntary with informed consent. Ethics approval was granted by the Institutional Review Board of The University of Hong Kong/Hospital Authority Hong Kong West Cluster (Reference Number: UW20-298). The study was registered with the National Institutes of Health (Identifier Number: NCT04365361).

### Recruitment Procedures

The online questionnaire was distributed *via* an anonymous link with an exponential, non-discriminative snowball sampling strategy (21), and set up so that browser cookies would prevent respondents from taking the survey a second time using the same browser. Such a design can prevent duplicate responses. Upon completion of the questionnaire, respondents received automatically computed scores with brief interpretations and explanations for scales included in the questionnaire to promote mental health awareness. Respondents did not receive any incentives, but were instead provided the contact numbers and links for seeking help, support, or further reliable information on COVID-19 (e.g., link to the World Health Organization website). A self-administered, anonymous questionnaire was used to collect students' demographic information and experiences in the UK (the country of study) during 1 January to 31 March 2020 (the initial stage of the pandemic). As of 31 March 2020, UK had around 3,000 confirmed COVID-19 cases, while Hong Kong had 68 cases (in which 17 were imported from other countries) (6, 7).

Four one-hour online focus group interviews were conducted with students who completed the survey and agreed to join the interviews on 3 May, 8 May (2 sessions), and 12 May 2020, moderated by the lead researcher (AYKL), a University academic, behavioral scientist and registered nurse with doctoral degrees in nursing and public health and 25 years of clinical nursing, teaching and research experience. A research assistant with a master's degree in psychology was responsible for taking notes during the interviews to record important points mentioned by interviewees. Another research assistant with a master's degree in sociology was responsible to monitor participants' response to ensure all participants actively participated in the interviewees.

The qualitative interviews were tape-recorded and transcribed verbatim. Questions were structured chronologically to aid recall and were phrased to provide scope for additional areas to emerge. The questions focused on students' experiences in the U.K. in relation to COVID-19, particularly with facemask wearing, and their stress and related stressors.

### Measurement

#### Academic Program Characteristics

Respondents were asked to indicate whether (i) they were studying full-time or part-time, (ii) they were final year students, (iii) their academic program included a practicum placement component, and (iv) their program was medical or health related. We did not ask in which University that students were studying. Asking such information could also be too sensitive as some students might not want to place blame on their universities. Hence, this study included students from different universities in the U.K.

#### Perceived Public Attitudes and Personal Belief and Practice in Relation to Facemask Wearing

Respondents were asked three questions regarding facemask wearing as a preventive measure against COVID-19. The first question was on their perception regarding the public's attitudes: “What was the public's attitude toward wearing facemasks as a preventive measure against COVID-19 in the U.K.?” Responses included two answer options: “viewed as preventive” and “did not view as preventive.” The second was on their personal beliefs regarding facemask wearing: “Did you believe that wearing a facemask was an effective preventive measure against COVID-19 in the U.K.?” Answer options were: “yes” and “no.” The third was on their practice of facemask wearing in public: “How often did you wear a facemask as a preventive measure against COVID-19 when going out in public in the U.K.?” Responses were made on a five-point Likert scale: “1 = never or almost never,” “2 = less than half of the time,” “3 = around half of the time,” “4 = more than half of the time,” and “5 = almost always or always.”

#### Stress in Relation to Facemask Wearing and Facemask-Related Stressors

Respondents were asked to recall the situation in their country of study from 1 January 2020 until 31 March 2020. Two questions asked about their feelings of stress from (i) wearing facemasks in public, “Did you ever feel stressed because you were wearing a facemask as a preventive measure against COVID-19?” and (ii) not wearing facemasks in public, “Did you ever feel stressed because you were not wearing a facemask when you went out in public?” Answer options were: “Yes” and “No.”

Another question was asked regarding stressors in relation to wearing facemasks (facemask-related stressors), “What factors do you believe contributed to your stress in relation to wearing a face mask as a preventive measure against COVID-19 when you were in the U.K.?” Answer options were: “public prejudiced attitudes against facemask wearing,” “social norms against facemask wearing,” “difficulties acquiring facemasks” and “high cost of facemasks.” Respondents could choose more than one answer option.

### Statistical Analysis

All statistical analyses were performed with SPSS for Windows (version 23.0). Chi-squared-test was used to examine the differences in demographics and other variables between students who joined the focus group interviews and those who did not join. All tests were two-sided, with *P* < 0.05 indicating statistical significance.

Logistic regression was used to examine (i) the differences in perceived public attitudes toward facemask wearing as a preventive measure against COVID-19, (ii) personal belief and practice in relation to facemask wearing; (iii) facemask-related stressors (social norms, prejudiced attitudes, difficulties acquiring facemasks and high cost of facemasks) between males and females, adjusted for potential confounders. Potential confounders included age group (18–25 years vs. 25 years or older), education program level (undergraduate vs. post-graduate), program with practicum component (yes vs. no), program year (final year vs. non-final year), and field of study (medical or health-related vs. others).

The interviews were audio-recorded and transcribed verbatim. The transcripts were analyzed by content analysis following the guidelines recommended by Morse and Field (22). Two researchers (AYKL and TTKL) independently reviewed and summarized the interview materials, including transcripts and recordings, and extracted meaningful statements. Field notes were reviewed during the analysis process. Conflicting opinions on the contents of a theme were discussed and resolved by a research group composed of three registered nurses (CHMK, JYYC, AYKL). Member checking, a respondent validation, was conducted by asking participants to review the transcripts from interviews they participated in and give feedback about emerging interpretations to ensure good representations of their realities, which helps to enhance the credibility of results. The software NVivo 11.0 (QSR International; Melbourne, VIC, Australia) was used to assist qualitative data administration, including creating codes, organizing, and summarizing data, searching for interrelationships between codes, and suggesting themes. We used a mixed-methods triangulation design to corroborate the findings of qualitative and quantitative data (23).

## Results

### Recruitment

**Figure 1:**
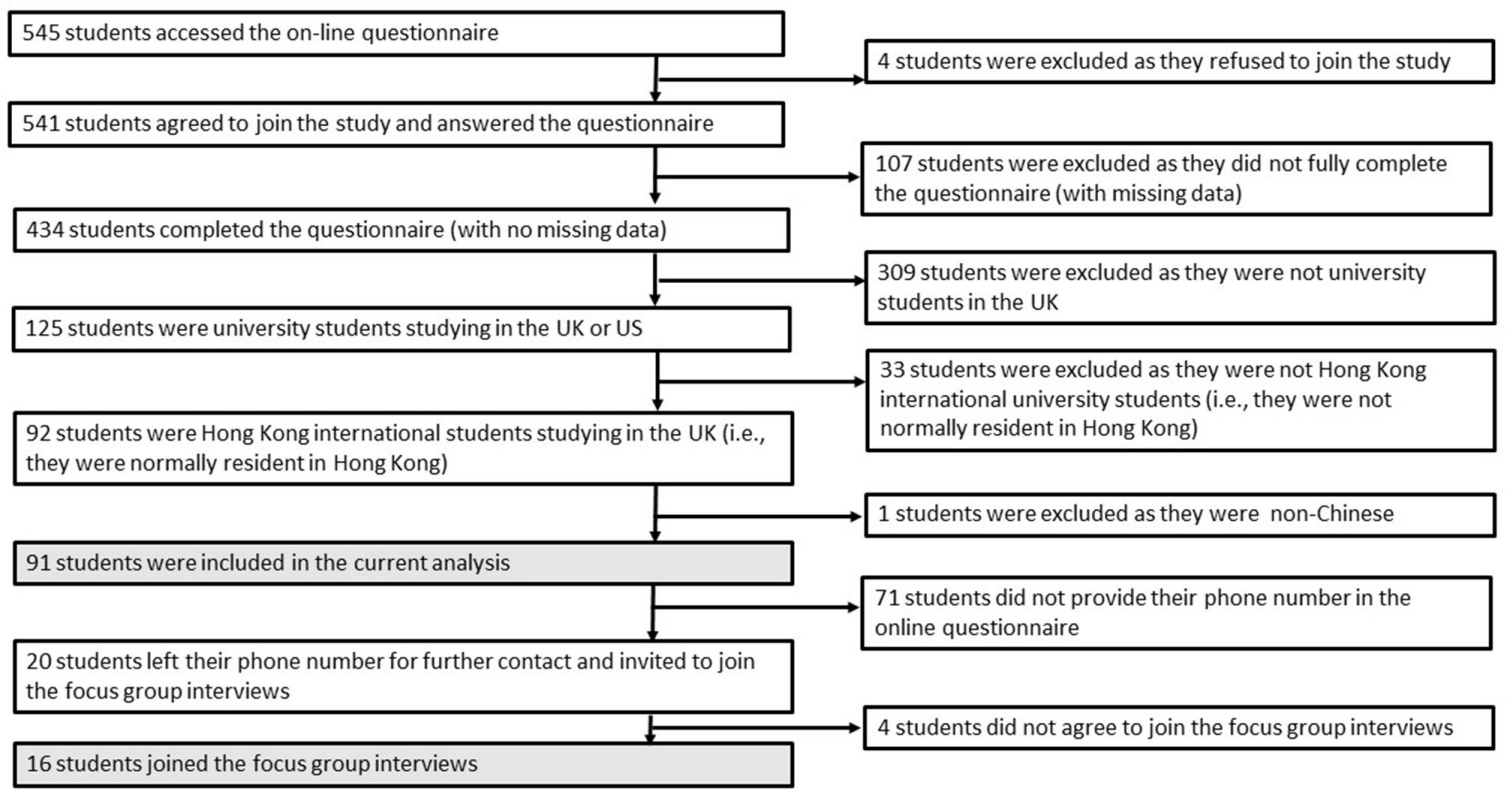
Flow diagram of the survey recruitment process.

Five hundred and forty-five respondents accessed the online survey from 28 April through 12 May 2020. All were University students. Four respondents refused to join. Five hundred and forty-one respondents accessed the link and agreed to join the online survey. One hundred and seven respondents did not provide complete data and were treated as missing values. Three hundred and nine respondents were not studying in the U.K., 33 respondents did not normally reside in Hong Kong, and one respondent was not Chinese. These respondents did not meet the inclusion criteria for this analysis and were excluded. The current analysis included 91 respondents, who were full-time Chinese students from Hong Kong studying in the UK who completed the online survey. Twenty respondents provided their contact number in the questionnaire and were invited to join the focus group interviews, of which 16 joined. Figure 1 shows the recruitment flow chart.

### Participants

Table 1 shows the 91 students (38.5% male and 93.4% aged 18–25 years) included in the study. 87.9 and 35.2% of them were undergraduates and in their final year, respectively. 58.2% were studying programs with a practicum component and 58.2% were studying a medical or health-related field. Twenty of these students provided their phone number and were contacted and invited to join the focus group interviews *via* WhatsApp. Four students refused to join. Sixteen students (37.5% male and all aged 18–25 years) joined the interviews. 68.8% were undergraduates and 37.5% were in their final year. 37.5% were studying programs with a practicum component, and 50% were pursuing a medical or health-related field. Greater proportion of students studying post-graduate program joined the interviews than those who did not (31.3 vs. 8.0%, *P* = 0.01).

**Table 1:** Characteristics of international students studying in the UK.

	**All *n* = 91**	**Did not join focus group *n* = 75**	**Joined focus group *n* = 16**	***P-*value**
	***n* (%)**	***n* (%)**	***n* (%)**	
**Sex**				
Males	35 (38.5)	39 (38.7)	6 (37.5)	
Females	56 (61.5)	46 (61.3)	16 (62.5)	0.93
**Age group**				
18–25 years	85 (93.4)	69 (92.0)	16 (100)	
25 years or older	6 (6.6)	6 (8.0)	0 (0)	0.59
**Education program level**				
Undergraduate	80 (87.9)	69 (92.0)	11 (68.8)	
Post-graduate	11 (12.1)	6 (8.0)	5 (31.3)	0.01^*^
**Program year**				
Non-final year	59 (64.8)	49 (65.3)	10 (62.5)	
Final year	32 (35.2)	26 (34.7)	6 (37.5)	0.46
**Program with practicum component**				
No	38 (41.8)	28 (37.3)	10 (62.5)	
Yes	53 (58.2)	47 (62.7)	6 (37.5)	0.06
**Field of study**				
Non-medical or health-related	38 (41.8)	30 (40.0)	8 (50.0)	
Medical or health-related	53 (58.2)	45 (60.0)	8 (50.0)	0.32

*Chi-square-test was performed*,

* *P < 0.05*.

### Perceived Public Attitudes and Personal Beliefs and Practice in Relation to Facemask Wearing

Table 2 shows 92.3% of the students reported that the public did not view facemask wearing as a preventive measure against COVID-19. Nearly all (98.9%) believed facemask wearing was an effective preventive measure against COVID-19, but only 56% wore a facemask more than half of the time when out in public. No significant difference in the above was found between males and females.

**Table 2:** Perceived public attitudes, and personal belief and practice in relation to facemask wearing as a preventive measure against COVID-19 in the UK.

	**All *n* = 91**	**Males *n* = 35**	**Females *n* = 56**	**Relative risk estimate (95%CI)**	***P-*value^a^**
	***n* (%)**	***n* (%)**	***n* (%)**		
**Perceived public attitudes toward wearing facemasks as a preventive measure**					
Viewed as preventive	7 (7.7)	4 (11.4)	3 (5.4)		
Did not view as preventive	84 (92.3)	31 (88.6)	53 (94.6)	1.07 (0.93, 1.22)	0.30
**Personal belief toward wearing a facemask as an effective preventive measure**					
No	1 (0.1)	1 (2.9)	0 (0.0)		
Yes	90 (98.9)	34 (97.1)	56 (100)	1.03 (0.97, 1.09)	1.00
**Personal practice of wearing a facemask when going out in public in the UK**					
Less than half of the time	40 (44.0)	19 (54.3)	32 (57.1)		
More than half of the time	51 (56.0)	16 (45.7)	24 (42.9)	0.94 (0.59, 1.50)	0.81

a *Logistic regression with adjustment of age group, education program level, program year, practicum component and field of study. No variables with missing data were noted*.

In the focus group interviews, students reported great differences in public attitudes toward facemask wearing as a preventive measure between the U.K. and H.K. They expressed their worries and fears on such differences and reported internal conflicts between what they knew (facemask wearing is an effective preventive measure) and what they were expected to do (not wear facemasks in public and in hospitals). They also reported frustrations with trying to persuade local students (their schoolmates) to wear facemasks. Some of their words are quoted below:

“*I wasn't allowed to wear a mask in the hospital for my placement… they thought it would create panic and fear. I really think their views on facemask wearing is very different than our views. I didn't think it was reasonable, but I needed to follow the instructions from my supervisors.” (Female, aged 22 years, an undergraduate student studying a medical and health-related programme)*

“*My friends didn't care about wearing facemasks, it was like normal life for them. When I explained that facemasks can stop you from spreading to other people, they said sick people should just stay home… I told them this would only happen in an ideal world and that's why facemasks are important. They said they weren't scared of the virus and it wasn't a matter of concern. Finally, I gave up trying to persuade them.” (Male, aged 21 years, an undergraduate student studying a non-medical and health-related programme)*

“*I had to explain to my teachers why I was worried, especially with my experience from SARS… my teachers thought we were overreacting when we wore facemasks in class… I offered masks to my U.K. friends, but they said they didn't need them. I felt like no one took it seriously.” (Female, aged 21 years, an undergraduate student studying a medical and health-related programme)*

“*The government repeatedly told us not to wear facemasks and not to worry… made me scared since I know wearing one is an effective preventive method.” (Female, aged 21 years, an undergraduate student studying a medical and health-related programme)*

### Stress in Relation to Facemask Wearing, Facemask-Related Stressors, and Coping Methods

Table 3 shows that 68.1% of the students reported feeling stressed when they wore facemasks out in public, with no difference between males and females. Regarding not wearing facemasks out in public, 62.6% felt stressed, significantly more females than males [(76.8 vs. 40.0%), *P* = 0.002, Relative Risk (RR), 95% confidence interval (C.I.): 1.92 (1.25, 2.95)]. 50.5% felt stressed both when wearing and not wearing facemasks out in public, significantly more females than males [76.8 vs. 40.0%, *P* = 0.004, RR (95% CI): 1.99 (1.17, 3.38)]. 61.5 and 56% of students reported prejudiced attitudes and social norms against facemask wearing that contributed to their stress when they were in the U.K., respectively. More females reported such prejudiced attitudes [71.4 vs. 45.7%, *P* = 0.02, RR (95% CI): 1.56 (1.05, 2.32)] than males.

**Table 3:** Stress in relation to facemask wearing and facemask-related stressors in the total student sample and subgroups.

	**All *n* = 91**	**Males *n* = 35**	**Females *n* = 56**	**Relative risk estimate (95%CI)**	***P-*value^a^**
	***n* (%)**	***n* (%)**	***n* (%)**		
**STRESS IN RELATION TO FACEMASK WEARING AS A PREVENTIVE MEASURE AGAINST COVID-19**					
**Felt stressed when wearing a facemask in public**					
No	29 (31.9)	15 (42.9)	14 (25.0)		
Yes	62 (68.1)	20 (57.1)	42 (75.0)	1.31 (0.95, 1.81)	0.11
**Felt stressed when not wearing a facemask in public**					
No	34 (37.4)	21 (60.0)	13 (23.2)		
Yes	57 (62.6)	14 (40.0)	43 (76.8)	1.92 (1.25, 2.95)	0.002^**^
**Felt stressed when both wearing and not wearing a facemask in public**					
No	45 (49.5)	24 (68.6)	21 (37.5)		
Yes	46 (50.5)	11 (31.4)	35 (62.5)	1.99 (1.17, 3.38)	0.004^**^
**FACEMASK-RELATED STRESSORS**					
**Prejudiced attitudes**					
No	35 (38.5)	19 (54.3)	16 (28.6)		
Yes	56 (61.5)	16 (45.7)	40 (71.4)	1.56 (1.05, 2.32)	0.02^*^
**Social norms**					
No	40 (44.0)	20 (57.1)	20 (35.7)		
Yes	51 (56.0)	15 (42.9)	36 (64.3)	1.50 (0.98, 2.30)	0.064
**Difficulties acquiring facemasks**					
No	61 (67.0)	27 (77.1)	34 (60.7)		
Yes	30 (33.0)	8 (22.9)	22 (39.3)	1.72 (0.86, 3.43)	0.07
**High costs of facemasks**					
No	75 (82.4)	31 (88.6)	44 (78.6)		
Yes	16 (17.6)	4 (11.4)	12 (21.4)	1.88 (0.66, 5.36)	0.22

a *Logistic regression with adjustment of age group, education program level, program year, practicum component and field of study*;

* *P < 0.05*,

** *P < 0.01*.

In the focus group interviews, students reported that they felt stressed and embarrassed when wearing facemasks and experienced prejudice and perceived to be stigmatized by others. Some of their words are quoted below.

“*No one really wore masks where I lived, but fortunately I never saw people purposely looking at me, unlike in London where violent attacks have happened… even though I felt relatively safe to wear a mask, I felt embarrassed to do so.” (Male, aged 20 years, an undergraduate student studying a non-medical and health-related programme)*

“*A few guys saw me and my friends wearing masks and purposely walked over and coughed in our faces… same thing happened with another man who started coughing really hard when he saw us walk near him to get a cab.” (Female, aged 21 years, an undergraduate student studying a non-medical and health-related programme)*

“*In February, while my friends and I were walking down the street wearing facemasks, someone yelled ‘coronavirus!' from across the street, labeling us as the virus.” (Female, aged 21 years, an undergraduate student studying a medical and health-related programme)*

“*While going into a restaurant for dinner, someone at the door asked where I was from… when I replied Hong Kong, they immediately said, ‘So do I need to wear a mask?' and proceeded to cover their nose and mouth with their hand.” (Male, aged 21 years, an undergraduate student studying a non-medical and health-related programme*)

However, students also reported some positive experiences from peer and family support. Positive thinking and adaptability were reported as effective methods of stress management.

“*I went to a number of pharmacy stores, but no facemasks were available. I tried to make online orders, but everything was out of stock… Finally, my mother sent boxes to me by express mail.” (Female, aged 21 years, an undergraduate student studying medical and health-related programme)*

“*One of my classmates shared some facemasks with me… and I shared my disinfectants with her.” (Male, aged 20 years, an undergraduate student studying a non-medical and health-related programme)*

“*I wore a facemask to protect myself when I was in the subway… but took it off and kept it in an envelope when I worked in the hospital. because I didn't want to act against the suggestions (instructions) from my supervisor … I put it (the facemask) on again when I was off duty.” (Female, aged 21 years, an undergraduate student studying medical and health-related programme)*

“*I'm proud of myself for being resourceful… when I couldn't buy any face masks, I used my scarf and turned it into a face covering.” (Female, aged 21 years, an undergraduate student studying a non-medical and health-related programme)*

“*I experienced some negative incidents (being yelled at when I wore a facemask in the supermarket) … had negative thoughts… but I'm not going to dwell on them. Life must go on. I tried to shift my thinking to be more positive.” (Male, aged 21 years, an undergraduate student studying a non-medical and health-related programme)*

## Discussion

This is the first report on stress associated with facemask wearing during COVID-19 among international students studying abroad. More than 90% of students from Hong Kong studying in the U.K. reported that the U.K. public did not view facemask wearing as a preventive measure against COVID-19 during the early stage of the pandemic. Contrasting public attitudes and personal beliefs might explain the stress from facemask wearing. More females reported perceived prejudiced attitudes or behaviors of others resulting in their stress in relation to facemask wearing. Half of the students were stressed about both wearing and not wearing facemasks in public. Peer and family support, positive thinking and adaptability were important for stress management.

Hong Kong has had previous experiences from the severe acute respiratory syndrome (SARS) outbreak in 2003. This might explain the positive beliefs and practice of H.K. international students regarding facemask wearing.

In the early stage of the COVID-19 pandemic when our survey was conducted, facemask wearing had been controversial with conflicting guidelines from different agencies and organizations (3, 24, 25). People from East Asia had a more positive view toward countermeasures to fight the pandemic, however, people from Western countries showed an unwillingness to learn from East Asian countries on handling such outbreaks (26). Besides, western countries had low perceived benefits of wearing facemask as self-protection and protecting others (27). These inconsistencies and discrepancies exacerbated non-compliance and rejection of facemasks, mostly in Western countries, as an essential tool against the pandemic. The sight of facemasks had become a catalyst for panic and violent attacks on Asians in many Western cities, where facemask wearing was less common and perceived as only worn by sick individuals (2, 3). These might explain why most Hong Kong students felt stressed about wearing facemasks and why many left the U.K. to return home with universal facemask wearing and a more controlled outbreak situation.

Significantly more female students reported stress related to facemask wearing and being prejudiced against. This is consistent with studies showing higher post-traumatic stress symptoms related to the pandemic (28) and increased vulnerability from exposure to persistent stress in females (29). With xenophobic and racist behaviors often rooted in fear, ignorance and a lack of awareness, and fueled by misinformation (1, 30, 31), schools and governments have the responsibility to communicate accurately and effectively with students and the public to mitigate these problems and negative consequences, especially from facemask wearing.

Fear and stress from xenophobic attacks compounded with physical threats of the pandemic can severely impact the mental well-being of University students. During the COVID-19 pandemic, Chinese International students have been faced with a difficult, confusing, and sensitive situation, and they require support from global communities and deserve to be treated fairly in their pursuit of quality education overseas. With more students set to gradually return to their respective schools of study, our findings have significant implications on the proactive roles that academic institutions and mental health professionals need to take to provide additional support for international students.

Positive thinking and adaptability were identified as important coping methods associated with less severe mental health impacts. Similar findings were reported in our sister paper (32). Online mindfulness-based interventions and mental health programs may help enhance students' resilience (33).

In future research, follow-up studies are needed to explore and monitor the impact of stress and prejudice experienced by international students during and after the COVID-19 pandemic. More research on the impact of other countermeasures against the pandemic (such as social distancing) and change in learning format on student mental health, and particularly intervention studies to enhance students' psychological resilience and positive coping are needed.

Our study had a few limitations. Firstly, because validated questionnaires were not available for measuring stress from specific stressors, we developed our own outcome-based questionnaire to assess beliefs, attitudes, and practice related to facemask wearing. The quantitative findings were corroborated by the qualitative findings from focus group interviews. Secondly, all the respondents were Chinese students from Hong Kong, and the sample size was small. Our results might not be generalizable to all Chinese international students in the UK and from other countries. Further studies, especially multi-country studies on international students using standardized methods are needed. Thirdly, we asked respondents to recall their experiences from January to March 2020, which might be subject to recall errors. Fourthly, the snowball sampling was an effective and efficient strategy to recruit respondents and collect valuable data at the height of the pandemic, but could have led to sampling bias from respondents forwarding the survey to peers with similar traits and characteristics (21). Lastly, the proportions of students who answered the online survey was higher in females than males. It might be because females are more likely to engage in online activity characterized by communication and exchanging of information whereas males are more likely to engage in online activity characterized by information-seeking (34). Online survey response behavior is a process of online information-exchange and online information-seeking (35). In addition, female college students responding at much higher rates than male students is a common phenomenon (36).

## Conclusions

To conclude, a majority of Hong Kong international University students studying in the UK perceived to be stigmatized and experienced prejudice. Chinese international students have been faced with a difficult, confusing, and sensitive situation. Owing to the ongoing pandemic, rising xenophobia and racist behaviors, and disruptions in students' studies in the U.K., the government should play proactive roles to provide clear and accurate public health information and conduct positive media promotion as health advocacy to change public attitudes and mitigate prejudice. Academic institutions and mental health professionals need to identify such problems and proactively provide additional support for Chinese international students.

## Data Availability Statement

The dataset presented in this article is not readily available because the sharing of data to third parties was not mentioned in subject's consent. Requests to access the dataset can be done by directly contacting the corresponding author.

## Ethics Statement

The studies involving human participants were reviewed and approved by The Institutional Review Board of The University of Hong Kong/Hospital Authority Hong Kong West Cluster (Reference Number: UW20-298). The patients/participants provided their written informed consent to participate in this study.

## Study Registration

The research protocol was registered at the National Institutes of Health (https://clinicaltrials.gov/) (identifier number: NCT04365361).

## Author Contributions

AL led the conception, design of the survey, and carried out the survey. AL and SS were responsible for interpreting the data and drafting the manuscript. AL and T-hL were involved in statistical analysis. AL, SS, TL, M-pW, CK, JC, YF, MI, and T-hL were closely involved in data interpretation and manuscript revision. All authors read and approved the final manuscript.

## Conflict of Interest

The authors declare that the research was conducted in the absence of any commercial or financial relationships that could be construed as a potential conflict of interest.
